# Impact on glucometric variables and quality of life of the advanced hybrid closed‐loop system in pediatric and adolescent type 1 diabetes

**DOI:** 10.1111/1753-0407.13426

**Published:** 2023-06-19

**Authors:** Alfonso Lendínez‐Jurado, Ana Gómez‐Perea, Ana B. Ariza‐Jiménez, Leopoldo Tapia‐Ceballos, Icía Becerra‐Paz, María F. Martos‐Lirio, Fernando Moreno‐Jabato, Isabel Leiva‐Gea

**Affiliations:** ^1^ Department of Pediatric Endocrinology Regional University Hospital of Malaga Málaga Spain; ^2^ Universidad de Málaga, Andalucía Tech Málaga Spain; ^3^ Instituto de Investigación Biomédica de Málaga (IBIMA) Málaga Spain; ^4^ Department of Pediatric Endocrinology Reina Sofia University Hospital Córdoba Spain; ^5^ Instituto Maimónides de Investigación Biomédica de Córdoba (IMIBIC), Universidad de Córdoba Córdoba Spain; ^6^ Servicio de Supercomputación y Departamento de Arquitectura de Computadores Universidad de Málaga Málaga Spain

**Keywords:** pediatric type 1 diabetes, advanced hybrid closed‐loop, time in range, time in hyperglycemia, MiniMed 780G, 儿童1型糖尿病, 高级混合闭环, 目标范围内时间, 高血糖时间, MiniMed^TM^ 780G

## Abstract

**Background:**

In recent years, technological advances in the field of diabetes have revolutionized the management, prognosis, and quality of life of diabetes patients and their environment. The aim of our study was to evaluate the impact of implementing the MiniMed 780G closed‐loop system in a pediatric and adolescent population previously treated with a continuous subcutaneous insulin infusion pump and intermittent glucose monitoring.

**Methods:**

Data were collected from 28 patients with type 1 diabetes aged 6 to 17 years, with a follow‐up of 6 months. We included both glucometric and quality of life variables, as well as quality of life in primary caregivers. Metabolic control variables were assessed at baseline (before system change) and at different cutoff points after initiation of the closed‐loop system (48 hours, 7 days, 14 days, 21 days, 1 month, 3 months, 6 months).

**Results:**

Time in range 70–180 mg/dL increased from 59.44% at baseline to 74.29% in the first 48 hours after automation of the new system, and this improvement was maintained at the other cutoff points, as was time in hyperglycemia 180–250 mg/dL (24.44% at baseline to 18.96% at 48 hours) and >250 mg/dL (11.71% at baseline to 3.82% at 48 hours).

**Conclusions:**

Our study showed an improvement in time in range and in all time spent in hyperglycemia from the first 48 hours after the automation of the system, which was maintained at 6 months.

## Graphical abstract

**Highlights**

- This study is the first to evaluate the efficacy of the closed‐loop system MiniMed 780G in children aged 6 to 17 years with type 1 diabetes previously receiving continuous subcutaneous insulin infusion and intermittent continuous glucose monitoring.
- This shows early results, with improvement in time in range and all hyperglycemia times, from the first 48 hours after system switch and for 6 months of follow‐up.
- There was an improvement in the Pittsburgh Sleep Quality Index score at 3 months, although it was not significant compared to the baseline value, with a high percentage of satisfaction with the new system.

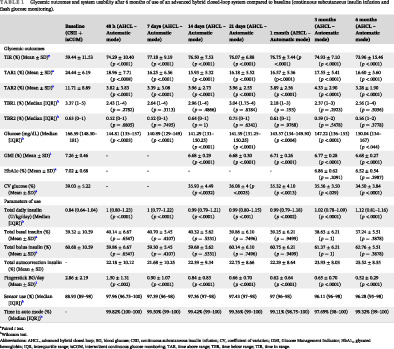

## 摘要

背景:近年来, 糖尿病领域的技术进步使糖尿病患者及其所处环境的管理、预后和生活质量发生了革命性的变化。本研究的目的是评估MiniMed^TM^ 780G闭环系统对既往接受过持续皮下胰岛素输注泵和间歇性血糖监测治疗的儿童和青少年人群的影响。

方法:收集28例年龄6~17岁的1型糖尿病患者资料, 随访6个月。我们纳入了血糖测定和生活质量变量, 以及主要照顾者的生活质量。在引入闭环系统后的不同时间点(48小时、7天、14天、21天、1个月、3个月、6个月)评估了代谢控制变量。

结果:在新系统自动化后的48小时内, 血糖在70~180 mg/dL范围内的时间从基线时的59.44%增加到74.29%, 并且这种改善在其他切点也是如此, 180~ 250mg/dL的高血糖时间(基线时24.44%降低到48小时的18.96%)和>250 mg/dL的高血糖时间(基线时11.71%降低到48小时时3.82%)也是如此。

结论:我们的研究表明, 从系统自动化后的最初48小时开始, 血糖控制范围内的时间和高血糖持续时间均有所改善, 并维持至6个月。

## 1 INTRODUCTION

Continuous glucose monitoring systems using intermittent or flash glucose monitoring (isCGM) measure glucose concentrations in interstitial fluid with subcutaneous electrodes, which are scanned by the patient or caregiver using a reader or cell phone. These monitoring systems are combined with multiple daily doses of insulin (MDI) or continuous subcutaneous insulin infusion (CSII) systems. However, the integration of real‐time continuous glucose measurement (rtCGM) data with continuous insulin infusion systems has radically changed the therapeutic approach to type 1 diabetes mellitus (T1D) by providing a major change in the proportion of patients achieving glycemic control targets. Glycemic targets for the pediatric population are set at >70% of the time between 70 and 180 mg/dL blood glucose (time in range: TIR), <25% above 180 mg/dL (time above range level 1: TAR1), <5% above 250 mg/dL (time above range level 2: TAR2), <4% below 70 mg/dL (time below range level 1: TBR1), and <1% below 54 mg/dL (time below range level 2: TBR2).^1^, ^2^

Multiple studies have shown that closed‐loop systems enable better glycemic control measured in glycated hemoglobin (HbA1c), TIR, hyperglycemia, hypoglycemia, lower variability, and improved quality of life.^3^, ^4^, ^5^, ^6^, ^7^, ^8^

One of the closed‐loop systems is the Medtronic MiniMed 780G device. It features an advanced hybrid closed‐loop (AHCL) algorithm that includes automatic delivery of basal insulin every 5 min, adjustable targets of 100 (5.50), 110 (6.10), and 120 (6.70) mg/dL (mmol/L), and automatic delivery of correction boluses every 5 min. Meal information must be input by the user or caregiver. Every 5 min autocorrection improves daytime blood glucose by mitigating inaccuracies in carbohydrate estimation and late or missed meal boluses and adapts to interday glucose variability without user intervention. This system was approved by the European Commission in June 2020 and is indicated for individuals with T1D aged 7 to 80 years, whose minimum total daily insulin dose is 8 U/day.^1^

Associated with the studies supporting the improvement of metabolic control with MiniMed 780G^1^, ^9^, ^10^, ^11^, ^12^ are cost‐utility and cost‐effectiveness studies that recognize the MiniMed 780G system as cost effective compared to isCGM plus MDI or CSII for the treatment of T1D.^13^, ^14^

Poor sleep quality is common in adolescents with T1D and their caregivers, probably secondary to fear of hypoglycemia, nocturnal glucose monitoring, hypoglycemia itself, and device alarms.^15^ Hybrid closed‐loop systems potentially reduce nighttime awakenings and fear of hypoglycemia and improve quality of life.^16^

Most of the published studies are in the adult population, with transitions from multiple doses to closed‐loop systems and in which the quality of life of the caregivers is not assessed. In this study, we evaluated the impact of the implementation of the MiniMed 780G closed‐loop system in a pediatric and adolescent population previously treated with CSII‐isCGM, studying both glucometric and quality of life variables as well as the quality of life of primary caregivers.

## 2 METHODS

This was a prospective, single‐center study carried out in the diabetes unit of a tertiary hospital in Spain (Regional University Hospital of Malaga) and based on data retrieved from digital medical records during the 6‐month follow‐up period. We included pediatric and adolescent patients aged 6 to 17 years, diagnosed with T1D, who were on combined treatment with CSII (MiniMed) and isCGM (FreeStyle Libre 2®) and had this system replaced by the MiniMed 780G AHCL system with Guardian 4 sensor (G4S) between December 2021 and April 2022. All the patients were followed by a multidisciplinary team comprising pediatric endocrinologists and diabetes nurses. Those with an interstitial glucose sensor system other than the one previously described were excluded.

Metabolic control variables were extracted using the LibreView and CareLink download platforms at the start of the study and at different cutoff points after reaching a daily time greater than 90% in auto mode (48 hours, 7 days, 14 days, 21 days, 1 month, 3 months, 6 months). Baseline sensor and insulin pump data were downloaded for the 2 weeks prior to the installation of the AHCL using the Libreview and Carelink System platforms, respectively. Once the closed‐loop system was initiated, data obtained during the first 48 hours, 7 days, and 14 days were downloaded using the CareLink System platform. After 21 days and at the following cutoff points (1 month, 3 months, 6 months), the data corresponding to the last 2 weeks were downloaded using the CareLink System platform.

The variables studied were:

*TIR: percentage of time in which interstitial blood glucose levels are between 70 and 180 mg/dL.

*TAR1: percentage of time in which interstitial blood glucose levels are between 180 and 250 mg/dL.

*TAR2: percentage of time in which interstitial blood glucose is above 250 mg/dL.

*TBR1: percentage of time in which interstitial blood glucose levels are between 70 and 54 mg/dL.

*TBR2: percentage of time in which interstitial blood glucose is below 54 mg/dL.

Other parameters included were the percentage of time in auto mode, coefficient of variation (CV), daily insulin dose (U/kg/day), percentage of insulin in basal or bolus form, the amount of insulin administered as automatic correction, Hb1Ac, Glucose Management Indicator (GMI), and mean glucose.

Quality of life variables (sleep and satisfaction with treatment) were also studied through questionnaires completed in person by the main caregivers both at baseline and 3 months after initiation of the closed‐loop system.

- Pittsburgh Sleep Quality Index (PSQI): The sum of the scores of the 19 questions, or the total score, indicates the overall sleep quality of the person being evaluated. This total score can range from 0 to 21 points. The higher the total score, the worse the sleep quality. Thus, a total score less than or equal to five on the Pittsburgh scale indicates that, in general, sleep quality is optimal, whereas a total score greater than five suggests sleep disturbances of greater or lesser severity.^17^
- Diabetes Treatment Satisfaction Questionnaire (DTSQ) index: assesses satisfaction (status version, DTSQ‐s) and change in satisfaction (change version, DTSQ‐c) with treatment for T1D. DTSQ‐s scores range from 0 to 48, and DTSQ‐c scores range from −24 to +24, with higher scores indicating greater satisfaction.^18^

The study protocol was in accordance with the Declaration of Helsinki. All participants and their caregivers were informed of the study and signed a consent form. The included one patient under 7 years who was 6 years and 6 months old. Parents were informed of the 6‐month difference in age limit for device use. The protocol was approved by the ethics committee of our center.

Data analysis was performed using free R 4.0.2 software (R‐CoreTeam 2020) (https://www.r-projetc.org/). A Shapiro‐Wilk test analysis was performed to determine the normality of the study variables. Results are presented as mean ± SD values in normal distributions or as median (interquartile range [IQR]) in nonnormal distributions. A Wilcoxon signed‐rank test was performed to analyze differences in the nonnormal distributions, and the paired *t* test was used in the normal distributions. Correlation analyses were performed using Pearson's correlation coefficient in parametric variables and Spearman's correlation coefficient in nonparametric variables. A chi‐square test was used to perform a bivariate analysis. *p*<.05 was considered statistically significant. *P* values were adjusted using the Benjamini‐Hochberg correction for multiple comparisons.

## 3 RESULTS

A total of 28 patients were included, 57% of whom were male. The mean age at the onset of diabetes was 4.16 ± 2.40 years (range 7 months–9 years and 3 months), and 12 ± 2.43 years (range 6 years and 6 months–16 years and 7 months) on initiation of the MiniMed 780G device. The mean time from onset to initiation of the closed‐loop system was 7.84 ± 2.46 years. The mean number of daily scans with CSII‐isCGM was 13.37 ± 9.41 scans/day. The mean daily sensor activation time was 88.93%, with a (IQR 89–98). Once the AHCL system was initiated, the daily activation time increased to 97.96% in the first 48 hours, reaching 99.5% after 7 days. The mean number of days from implementation of the closed‐loop device in manual mode until switching to auto mode (with a percentage of time per day greater than 90%) was 3.44 days (IQR 3‐4). Once this time was reached, at 48 hours, the system operated in auto mode 99.82% of the time (Table 1).

**TABLE 1:** Glycemic outcomes and system usability after 6 months of use of an advanced hybrid closed‐loop system compared to baseline (continuous subcutaneous insulin infusion and flash glucose monitoring).

	Baseline (CSII + isCGM)	48 h (AHCL – Automatic mode)	7 days (AHCL – Automatic mode)	14 days (AHCL – Automatic mode)	21 days (AHCL – Automatic mode)	1 month (AHCL – Automatic mode)	3 months (AHCL – Automatic mode)	6 months (AHCL – Automatic mode)
Glycemic outcomes								
TIR (%) (Mean ± SD)^a^	59.44 ± 11.53	74.29 ± 10.40 (*p* <.0001)	77.18 ± 9.19 (*p* <.0001)	76.50 ± 7.53 (*p* <.0001)	76.07 ± 6.88 (*p* <.0001)	76.75 ± 7.44 (*p* <.0001)	74.93 ± 7.10 (*p* <.0001)	73.96 ± 15.46 (*p* <.0001)
TAR1 (%) (Mean ± SD)^a^	24.44 ± 6.19	18.96 ± 7.71 (*p* <.0008)	16.25 ± 6.56 (*p* <.0001)	15.93 ± 5.32 (*p* <.0001)	16.18 ± 5.52 (*p* <.0001)	16.57 ± 5.36 (p<.0001)	17.55 ± 5.41 (*p* <.0001)	16.60 ± 5.60 (*p* <.0001)
TAR2 (%) (Mean ± SD)^a^	11.71 ± 8.89	3.82 ± 3.83 (*p* <.0001)	3.39 ± 3.08 (*p* <.0001)	3.96 ± 2.73 (*p* <.0001)	3.96 ± 2.55 (*p* <.0001)	3.89 ± 2.56 (*p* <.0001)	4.33 ± 2.90 (*p* <.0001)	3.28 ± 1.90 (*p* <.0001)
TBR1 (%) (Median [IQR])^b^	3.37 (1–5)	2.43 (1–4) (*p* = .2782)	2.64 (1–4) (*p* = .3113)	2.96 (1–4) (*p* = .4866)	3.04 (1.75–4) (*p* = .8184)	2.18 (1–3) (*p* = .193)	2.37 (1–3) (*p* = .2923)	2.56 (1–4) (*p* = .5056)
TBR2 (%) (Median [IQR])^b^	0.63 (0–1)	0.52 (0–1) (*p* = .6805)	0.52 (0–1) (*p* = .7495)	0.64 (0–1) (*p* = 1)	0.75 (0–1) (*p* = .6341)	0.61 (0–1) (*p* = .9768)	0.59 (1–2) (*p* = .5478)	0.56 (1–2) (*p* = .3778)
Glucose (mg/dL) (Median [IQR])^b^	166.59 (148.50–181)	144.81 (135–157) (*p* <.0003)	140.89 (129–149) (*p* <.0001)	141.29 (131–150.25) (*p* <.0001)	141.39 (131.25–150.25) (*p* <.0001)	143.57 (134–149.50) (*p* <.0004)	147.22 (136–153) (*p* <.0001)	150.04 (134–167) (*p* <.044)
GMI (%) (Mean ± SD)^1^	7.26 ± 0.46	‐	‐	6.68 ± 0.29 (*p* <.0001)	6.68 ± 0.30 (*p* <.0001)	6.71 ± 0.26 (*p* <.0001)	6.77 ± 0.28 (*p* <.0001)	6.68 ± 0.27 (*p* <.0001)
HbA1c (%) (Mean ± SD)^a^	7.02 ± 0.68	‐	‐	‐	‐	‐	6.86 ± 0.62 (*p* = .2091)	6.52 ± 0.54 (*p* = .2987)
CV glucose (%) (Mean ± SD)^a^	39.03 ± 5.22	‐	‐	35.93 ± 4.49 (*p* <.0032)	36.08 ± 4 (*p* <.0025)	35.32 ± 4.10 (*p* <.0013)	35.56 ± 3.50 (*p* <.029)	34.50 ± 3.84 (*p* <.0001)
Parameters of use								
Total daily insulin (U/kg/day) (Median [IQR])^b^	0.84 (0.64–1.04)	1 (0.80–1.23) (*p* <.0001)	1 (0.77–1.22) (*p* <.0001)	0.99 (0.79–1.21) (*p* <.0001)	0.99 (0.80–1.15) (*p* <.001)	0.99 (0.79–1.18) (*p* <.0002)	1.02 (0.78–1.09) (*p* <.0001)	1.12 (0.81–1.16) (*p* <.0001)
Total basal insulin (%) (Mean ± SD)^a^	39.32 ± 10.59	40.14 ± 6.67 (*p* = .6547)	40.70 ± 5.45 (*p* = .4107)	40.32 ± 5.62 (*p* = .5331)	39.86 ± 6.10 (*p* = .7496)	39.25 ± 6.21 (*p* = .9499)	38.63 ± 6.21 (*p* = 1)	37.24 ± 5.51 (*p* = .3878)
Total bolus insulin (%) (Mean ± SD)^a^	60.68 ± 10.59	59.86 ± 6.67 (*p* = .6547)	59.30 ± 5.45 (*p* = .4107)	59.68 ± 5.62 (*p* = .5331)	60.14 ± 6.10 (*p* = .7496)	60.75 ± 6.21 (*p* = .9499)	61.37 ± 6.21 (*p* = 1)	62.76 ± 5.51 (*p* = .3878)
Total autocorrection insulin (%) (Mean ± SD)	‐	22.18 ± 10.12	21.68 ± 10.25	22.39 ± 9.34	22.75 ± 8.66	22.29 ± 8.64	23.93 ± 8.03	25.52 ± 8.55
Fingerstick BG/day (Mean ± SD)^1^	2.86 ± 2.19	1.50 ± 1.31 (*p* <.002)	0.90 ± 1.07 (*p* <.0001)	0.84 ± 0.83 (*p* <.0001)	0.66 ± 0.70 (*p* <.0001)	0.62 ± 0.64 (*p* <.0001)	0.65 ± 0.70 (*p* <.0001)	0.52 ± 0.29 (*p* <.0001)
Sensor use (%) (Median [IQR])^b^	88.93 (89–98)	97.96 (96.75–100)	97.39 (96–98)	97.36 (97–98)	97.43 (97–98)	97 (96–98)	96.11 (96–98)	96.28 (95–98)
Time in auto mode (%) (Median [IQR])^b^	‐	99.82% (100–100)	99.50% (99–100)	99.42% (99–100)	99.36% (99–100)	99.11% (98.75–100)	97.69% (98–100)	99.32% (99–100)

^a^ Paired *t* test.

^b^ Wilcoxon test.

Abbreviations: AHCL, advanced hybrid closed loop; BG, blood glucose; CSII, continuous subcutaneous insulin infusion; CV, coefficient of variation; GMI, Glucose Management Indicator; HbA1_c_, glycated hemoglobin; IQR, interquartile range; isCGM, intermittent continuous glucose monitoring; TAR, time above range; TBR, time below range; TIR, time in range.

The median total insulin before the system change was 0.84 IU/kg/day. At 48 hours we observed a mean increase of 0.16 IU/kg/day in the amount of insulin, with slightly differences in the other cutoff points. Our statistical analysis showed these increases were significant in all the cutoff points studied. The percentage of basal and bolus insulin was maintained with little difference despite the start of the closed loop, and no statistical significance was found in these variations. Once the closed‐loop system was initiated, we found that at 48 hours the percentage of self‐correction remained around 22%, increasing to 25% at 6 months (Table 1).

We calculated the mean glucose obtained with CSII‐isCGM and after initiation of the new system. Baseline values were 166.59 mg/dL, with a statistically significant decrease in mean values after AHCL implementation with respect to the baseline value. GMI with the initial system was 7.26%, with a decrease 14 days after initiation of the hybrid system to 6.68%. This figure remained stable at all other cutoff points, and these differences were statistically significant throughout the study period. Regarding CV, we found that at baseline, patients had a percentage of up to 39.03%. At 14 days, this figure was 35.93%, which was a statistically significant decrease in comparison with the baseline value. From this point onward, the CV value established by consensus (CV <36%) was achieved.^2^ This decrease remained stable at 1,3 and 6 months and continued to be statistically significant compared to the baseline value (Table 1).

When assessing TIR, we observed that before use of the AHCL system the baseline value was 59.44%. After initiation of the AHCL system, the mean TIR value obtained at 48 hours increased 19.85% compared to the baseline value. This increase remained stable throughout follow‐up and was statistically significant at all the cutoff points studied (Table 1). A correlation was seen when comparing TIR at 3 months with the percentage of autocorrection in this same period, showing an inversely proportional relationship to the percentage of TIR (R^2^ = 0.3695, *p* <0.0008). When dividing our patients into two groups, according to whether or not they met the target figures according to consensus (TIR >70%), we observed that prior to initiating the closed‐loop system, only 18.52% of the patients attained these values (Table 2). After 48 hours, we found an increase to 67.86%, reaching 78.57% at month. At 3 months we observed a nadir in the figures of up to 66.67%, which was maintained at 6 months at 68%. When comparing the different cutoff points with the baseline value, no significant differences were found in the percentage of patients.

**TABLE 2:** Percentage of patients meeting ATTD 2019 consensus criteria at the different cutoff points.

	Baseline (CSII + isCGM)	48 h (AHCL − Automatic mode)	7 days (AHCL − Automatic mode)	14 days (AHCL − Automatic mode)	21 days (AHCL − Automatic mode)	1 month (AHCL − Automatic mode)	3 months (AHCL − Automatic mode)	6 months (AHCL − Automatic mode)
TIR (% patients)	18.52	67.86 (*p* = .0798)	75 (*p* = .1428)	75 (*p* = .1428)	71.43 (*p* = .108)	78.57 (*p* = .1855)	66.67 (*p* = .0702)	68 (*p* = .6123)
TAR1 (% patients)	55.56	82.14 (*p* <.0057)	89.29 (*p* <.04)	100 (*p* = .5637)	96.43 (*p* = .2546)	92.86 (*p* = .1003)	92.59 (*p* = .9096)	96 (*p* = .2268)
TAR2 (% patients)	32.14	67.86 (*p* <.0172)	75 (*p* = .4304)	71.43 (*p* = .7325)	75 (*p* = .9432)	82.14 (*p* = .6014)	62.96 (*p* = .7798)	84 (*p* = .2059)
TBR1 (% patients)	70.37	89.29 (*p* = .8815)	89.29 (*p* = .5737)	78.57 (*p* = .5737)	78.57 (*p* = .2153)	85.71 (*p* <.0314)	96.30 (*p* = .5359)	84 (*p* = .2059)
TBR2 (% patients)	85.19	92.86 (*p* = .5399)	92.86 (*p* = .5399)	89.29 (*p* = .4436)	85.71 (*p* = .3662)	89.29 (*p* = .4436)	92.59 (*p* = .0764)	88 (*p* = .0934)

Abbreviations: ATTD, Advanced Technologies & Treatments for Diabetes; CSII, continuous subcutaneous insulin infusion; isCGM, intermittent continuous glucose monitoring; TAR, time above range; TBR, time below range; TIR, time in range.

With respect to TAR1, we noted that before the change of system the mean percentage was 24.44%. At 48 hours, the decrease was 5.48% and at 7 days was 8.19%, reaching a mean percentage of 16.25%, which remained significantly stable over the following 6 months (Table 1). When evaluating the relationship between the percentage of TAR at 48 hours and the percentage of autocorrection at the same time point, a directly proportional and significant correlation was observed (R^2^ = 0.3517, *p* <.0009), as well as at 3 months (R^2^ = 0.4063, *p* <.0004). Categorizing our patients into two groups, according to whether or not they met the target figures as per consensus (TAR1 <25%), we observed that prior to the implementation of the closed‐loop system, 55.56% of them had already achieved these values. Once this system was initiated we noted a progressive increase, with a percentage of patients at 82.14% in the first 48 hours and 89.29% at 7 days. After 14 days and at the following cutoff points, it was observed that more than 90% of patients met this goal (Table 2). However, statistically significant differences were found only at 48 hours (*p* <.0057) and at 7 days (*p* <.04).

Regarding TAR2, our patients previously presented a percentage of up to 11.71%. The mean TAR2 value obtained at 48 hours after the change of system decreased 7.89% compared to the baseline value (Table 1). From this point onwards, the consensus target was reached and this significant improvement remained stable for the rest of the cut‐off points. A directly proportional relationship was observed between the percentage of TAR2 at 3 months (R^2^ = 0.2033) and the percentage of autocorrection in that period (*p* <.0183). When we divided our patients into two groups according to whether or not they met consensus targets (TAR2 <5%), we found that with CSII‐isCGM, only 32.14% of our patients met the targets. After 48 hours of the placement of the new system, up to 67.86% of our patients already spent <5% of time in severe hyperglycemia, with similar figures being obtained in the rest of the points studied (Table 2). No statistically significant improvements were observed after initiation of the closed‐loop system.

The mean baseline percentage of TBR1 was 3.37%. When we compared TBR1 percentages in AHCL with the baseline percentage we found discrete changes at 48 hv, with a decrease in the mean TBR1 of 0.94%. This slight improvement was constant at all other cutoff points, with no statistically significant differences when compared to baseline (Table 1). When classifying the patients into two groups according to whether or not they met the objectives of good control according to consensus (TBR1 <4%), we noted that 70.37% of them already met these objectives at baseline. After the change of system, the percentage increased to 89.29% in the first 48 hours and remained unchanged at 7 days (Table 2). At this point discrete variations without statistical significance were observed when compared to baseline, except for the percentage obtained at one month, 85.71%.

When TBR2 was assessed, the baseline percentage was 0.63%. Comparing TBR2 percentages to the baseline percentage we found that at 48 hours, time in hypoglycemia was 0.52%, with a decrease of 0.11%. Similar changes were observed during follow‐up, with no statistically significant improvement after initiation of the closed‐loop system (Table 1). When dividing the patients into two groups according to whether or not they met the objectives of good control according to consensus (TBR2 <1%), 85.19% of them met this objective with CSII+isCGM. After the change of system, the percentage increased to 92.86% in the first 48 hours and was maintained similar during the follow‐up (Table 2). No statistically significant differences were observed when comparing the percentage of patients who were well controlled according to consensus at any of the different cutoff points studied.

The mean score for the DTSQ prior to the change of system (DTSQ‐s) was 35.92 (IQR 33.75–39). Three months after its implementation (DTSQ‐c), the mean score was 12.88 (IQR 11–15). The questionnaire on the sleep quality of both parents (PSQI) showed a baseline value of 6.50 (IQR 2–11.25) and 5 (IQR 3–5.50) 3 months after implementation of the closed‐loop system, although this difference was not statistically significant (*p* = .09448). When this variable was divided into two subgroups according to the score (≤5 optimal sleep quality, >5 poor night's rest), up to 50% of the parents did not have adequate rest at baseline. Three months after introduction of the new system, up to 70.33% had optimal sleep quality. However, this difference was not statistically significant (*p* = .5148).

## 4 DISCUSSION

This study is the first to evaluate the efficacy of the MiniMed 780G AHCL system in children aged 6 to 17 years with T1D previously receiving CSII and isCGM therapy. Recent studies have examined the performance of the MiniMed 780G in subjects who switched from MDI or open systems to AHCL but not from isCGM+CSII to the MiniMed 780G. We also provide earlier outcome assessment data than previous studies of AHCL implementation, showing benefits as early as 48 hours after system initiation.

Closed‐loop systems offer the maximum benefit in terms of TIR, versus isCGM with or without CSII, and can achieve TIRs of over 80% in studies in adults, with most of the remaining time in hyperglycemia (TAR1 and TAR2), especially postprandially.^19^, ^20^, ^21^, ^22^

In our study, we evaluated metabolic control in children with isCGM+CSII who switched to the Medtronic 780G system, according to the variables defined following the introduction of sensor‐based glucose monitoring, as in other studies with these characteristics. Our cutoffs for good control are the typical cutoffs used in other studies, differentiating in our case, as in Víbora et al, between the cutoffs 70–180 mg/dL (TIR), <70 mg/dL (TBR1), <54 mg/dL (TBR2), 180–250 mg/dL (TAR1), and >250 mg/dL (TAR2).^23^

In a literature review of the last 10 years, we found three studies of isCGM and CSII in adults, and one in children, comparing isCGM and CSII with Medtronic 640G and rtCGM (a model prior to the 780G analyzed in our study), which found no differences in TIR.^24^

A more recent study in adolescents conducted by Gros Herguido et al showed an increase in TIR (65.30% vs. 73.80%), a decrease in CV from 36% to 31.60%, and a decrease in TAR from 26.60% to 19.30% and a decrease in TBR from 4.60% to 2.30% 6 months after switching from isCGM+CSII to rtCGM+CSII Medtronic 780G.^25^ Massa et al showed a similar study with a young population (5–16 years) but without providing such early data. In addition, the change of system is made to the MiniMed 640G, a model inferior to the one proposed in our study.^26^ In their work, Massa et al analyzed the switch from isCGM to rtCGM in a prospective study of 20 children with T1D, for 6 months. The end points of the study were the level of metabolic control reflected by HbA1c, the mean glucose level, and the glucose variability assessed by the CV measured at 1 month, 3 months, and 6 months after the switch, with our study providing earlier data and with a more recent AHCL model. Another difference is that they provide the means of the variables but do not specify the percentage of patients who reach the desired objectives according to consensus.

Most studies in children describe the transition from multiple doses to rtCGM+CSII, reporting data on improvement in the latter with respect to the former, whereas those studies that describe the transition from isCGM+CSII to rtCGM+CSII refer to articles on Medtronic technology previous to the 780G, the 640G system, which showed improved results such as lower glycemic variability (CV 38.40% at 3 months and 36.40% at 6 months, *p* <.001; vs. 46.20% at baseline, *p* <.0001),^26^ and lower TBR2 (0.90% [IQR 0.40–1.55] vs. 5.60% [IQR 3.05–9.55, *p* <0.0001]).^27^ Interestingly, Telliam et al published similar times spent in TBR1 in both systems (640G vs. CSII+isCGM), versus Massa et al who did identify differences (1.60% at 3 months and 1.50% at 6 months vs. 7.40% at baseline, *p* <.0001).

The MiniMed 780G system provides new advantages, such as earlier improvement in metabolic control^28^ and adjustable targets, which improve results with respect to its predecessor.^11^ Studies published in the pediatric population using this technology show a higher percentage of TIR, such as that carried out by Petrovski et al when comparing MDI + CGM versus AHCL MiniMed 780G (42.10% ± 18.70% baseline vs. 78.80% ± 6.10% at 3 months [*p* <.001])^29^ or that by Bassi et al, which compared two AHCL systems (MiniMed 780G vs. Tandem‐IQ), with better results obtained with the Medtronic technology (TIR at 1 month 74.80% vs. 65.50%, respectively, *p* = .004).^30^ However, studies such as that of Seget et al show no changes in glycemic control once AHCL is initiated and after 1 year of follow‐up (TIR 79.28% ± 8.12% vs. 80.40% ± 8.25%, *p* >.05).^31^

With regard to these data, our study showed a significant improvement in TIR early after the change of system, which was also significant at all the cutoff points compared to baseline (Table 1). Previously, the mean TIR value of our patients did not meet the standardized values for this variable (TIR >70%),^2^ and this objective was reached in the first 48 hours from initiation of the closed‐loop system and was maintained throughout the evaluation through 6 months of follow‐up. When classified into two groups according to whether or not they met the TIR objective according to consensus, it was observed that after the implementation of the closed‐loop system, the percentage of patients achieving this objective at all cutoff points changed significantly compared to baseline, although this was not significant and there was a nadir at 3 months, which was maintained at 6 months (Table 2). This decrease can guide us in the need for educational support and reinforcement concerning the adjustment of modifiable variables of the system, such as lowering the target to cutoff points at 100 mg/dL, decreasing the duration of insulin and optimizing the adjustment of the ratio, avoiding food intake with bolus omission, or the overcorrection of hypoglycemia.

Although most published studies describe changes in TIR, CV, or HbA1c results, modifications in TAR1 or TAR2 after AHCL implementation are rarely reported. Forlenza et al showed an improvement in TAR >180 mg/dL from 41% ± 14.70% to 33% ± 9.90% (*p* <.001) at 6 months after AHCL implementation of the 670G in children,^32^ and Renard et al showed a decrease in TAR >180 mg/dL from 41.90% to 30% (*p* = .007),^33^ although they used a model prior to the 780G and do not distinguish between TAR1 and TAR2. For this reason, in our study we examined the changes in TAR, differentiating between the two levels. With regard to TAR1, our patients had already met the target figures established in the international guidelines with the CSII+isCGM system, with a mean percentage <25% (Table 1).^2^ Concerning the classification of our sample into two groups, according to whether or not they met target figures for TAR1 <25%, we noted that this percentage of patients remained stable over time once the closed‐loop system had been introduced, with the great majority of our patients meeting this target (Table 2).

In addition, with the CSII + isCGM system, the mean percentage of TAR2 was much higher than the figures established by consensus as good control (TAR2 <5%). After the change of system, we noted a drastic reduction in this percentage in all the cutoff points studied, meeting consensus targets after the first 48 hours. When classifying our patients according to whether or not they met the TAR2 <5% target figures, we found that this percentage of patients doubled 48 hours after initiation of AHCL. Despite having carried out a literature search on this, we found no information to corroborate these data in other publications, and therefore further studies are needed.

Moreover, after classifying our patients into two groups according to whether or not they met the consensus targets,^2^ we observed an inversely proportional relationship between TIR and TAR1 and TAR2 and the percentage of autocorrection, such that those patients who did not meet these targets had a higher percentage of autocorrection. This finding has not been described in published studies to date.

Regarding time in hypoglycemia, in our series, TBR1 (<4%) and TBR2 (<1%) targets were already being met according to consensus at baseline before the change of system.^2^

As regards the sleep quality of the caregivers in our study (PSQI), and as in other studies of similar characteristics that compared this variable, no significant differences were found after initiation of the closed‐loop system despite lower scores on the questionnaires.^15^ The number of alarms was not correlated with the sleep score, and 3‐month assessment could be early to find differences in the results. When assessing treatment satisfaction surveys (DTSQ), high scores were obtained with both CSII+isCGM and AHCL.

Barnard et al compared CSII and isCGM with CSII and rtCGM in 26 children during 12 weeks of use of closed‐loop technology, activating the auto mode at night and the manual mode during the day, and 12 weeks of open loop. Even though they did not have a continuous closed loop or auto mode as in our study, improvements in sleep and self‐esteem were reported when the system was changed.^34^

As limitations, differences in SD were not assessed during the follow‐up, carbohydrate ration were not included and a possible bias in baseline GMI due to higher adherence in the previous 14 days related to the change of system. The main limitations of our study could be the small number of patients, the comparison of glucose values from different sensors, (Freestyle Libre 2 at baseline and Guardian 4 sensor during follow‐up), and the short follow‐up time, which could be extended in the future. Nonetheless, to date there are no published studies in the pediatric population with a longer follow‐up period than ours, and none have provided data so early in the follow‐up.

## 5 CONCLUSION

In our study, the MiniMed 780G system was shown to be a useful tool from the time of its implementation to improve metabolic parameters early on compared to the isCGM system with CSII, especially time in hyperglycemia, in children and adolescents with T1D.

## CONFLICT OF INTEREST STATEMENT

No potential conflicts of interest relevant to this article were disclosed.
